# ^15^N-, ^13^C- and ^1^H-NMR Spectroscopy Characterization and Growth Inhibitory Potency of a Combi-Molecule Synthesized by Acetylation of an Unstable Monoalkyltriazene

**DOI:** 10.3390/molecules22071183

**Published:** 2017-07-19

**Authors:** Zhor Senhaji Mouhri, Elliot Goodfellow, Steven P. Kelley, Robin S. Stein, Robin D. Rogers, Bertrand J. Jean-Claude

**Affiliations:** 1 Cancer Drug Research Laboratory, Department of Medicine, Division of Medical Oncology, McGill University Health Center/Glen Hospital, 1001 Decarie Blvd., Montreal H4A 3J1, QC, Canada; zhor.senhajimouhri@mail.mcgill.ca (Z.S.M.); elliot.goodfellow@mail.mcgill.ca (E.G.); 2 Department of Chemistry, McGill University, 801 Sherbrooke St. W., Montreal H3A 0B8, QC, Canada; steven.kelley@mcgill.ca (S.P.K.); robin.stein@mcgill.ca (R.S.S.); robin.rogers@mcgill.ca (R.D.R.)

**Keywords:** ^15^N-NMR, 1,2,3-triazene, temozolomide, monoalkyltriazene, 4-amino-1,8-naphthalimide

## Abstract

6-(3-Methyltriaz-1-en-1-yl)-1*H*-benzo[de]isoquinoline-1,3(2*H*)-dione referred to as EG22 (**8a**), is an open-chain 3-alkyl-1,2,3-triazene termed “combi-molecule” designed to inhibit poly(adenosine diphosphate ribose) polymerase (PARP) and damage DNA. To delay its hydrolysis, acetylation of N3 was required. Being a monoalkyl-1,2,3-triazene, EG22 could assume two tautomers in solution or lose nitrogen during the reaction, thereby leading to several acetylated compounds. Instead, one compound was observed and to unequivocally assign its structure, we introduced isotopically labeled reagents in its preparation, with the purpose of incorporating ^15^N at N2 and ^13^C in the 3-methyl group. The results showed that the 1,2,3-triazene moiety remained intact, as confirmed by ^15^N-NMR, coupling patterns between the ^15^N-labeled N2 and the ^13^C-labeled methyl group. Furthermore, we undertook heteronuclear single quantum coherence (HSQC) and heteronuclear multiple bond correlation (HMBC) experiments that permitted the detection and assignment of all four nitrogens in 6-(3-acetyl-3-methyltriaz-1-en-1-yl)-1*H*-benzo[de]isoquinoline-1,3(2*H*)-dione, referred to as ZSM02 (**9a**), whose structure was further confirmed by X-ray crystallography. The structure showed a remarkable coplanarity between the N-acetyltriazene and the naphtalimide moiety. Thus, we unequivocally assigned **9a** as the product of the reaction and compared its growth inhibitory activity with that of its precursor, EG22. ZSM02 exhibited identical growth inhibitory profile as EG22, suggesting that it may be a prodrug of EG22.

## 1. Introduction

**Scheme 1:**
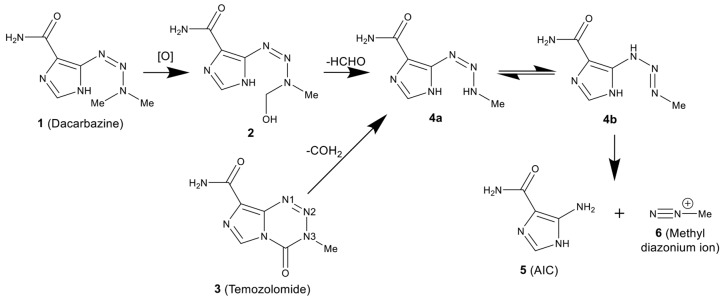
Metabolic pathway for dacarbazine and temozolomide [1,2,3,8].

The alkyltriazenes are amongst the oldest classes of alkylating agents used in the clinic for the treatment of cancer. One such agent, dacarbazine (**1**, Scheme 1), has been used in the clinical management of malignant melanoma for more than 35 years [1,2,3]. As depicted in Scheme 1, in vivo, dacarbazine (**1**) is metabolized into the monoalkyltriazene **4a** that is further hydrolyzed to give the methyl diazonium species that alkylates DNA [4]. It is also known to be the species released from the hydrolysis of Temodal, a potent clinical agent used in the clinical management of glioblastoma [5]. Monoalkyltriazene **4a** is in equilibrium with its corresponding tautomer **4b**, which upon hydrolytic cleavage, generates aminoimidazole carboxamide **5** and a methyldiazonium species **6** [6] that reacts with DNA in the cells to generate many adducted bases, including N3-methyladenine, N7-methylguanine, N7-methyladenine and *O*-6-methylguanine adducts [2,7,8].

**Scheme 2:**
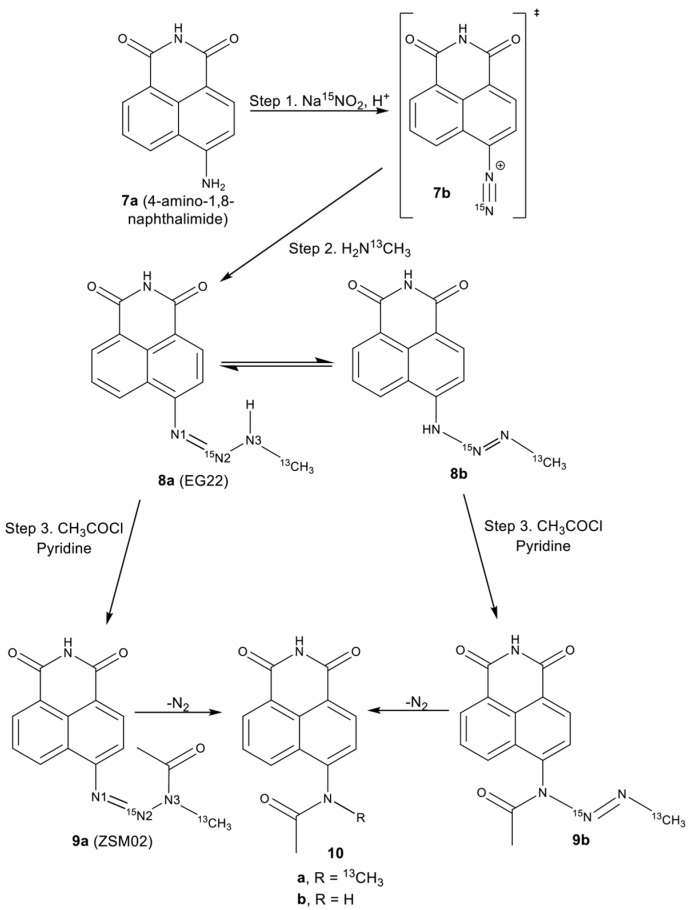
Synthesis of ZSM02 (**9a**) and possible by-products.

The mechanism of hydrolysis of monoalkyltriazenes of type **4a**, which leads to an aromatic amine (e.g., **5**) and a DNA alkylating agent, has inspired the development by our group of a new tumour targeting approach termed “combi-targeting” [9,10,11]. This approach seeks to design molecules termed “combi-molecules” to behave as bioactive species on their own and to further be hydrolyzed into other bioactive species. Agents that require hydrolysis to generate bioactive species are termed type I combi-molecules [12] and those that do not require hydrolysis to exert multiple activities, type II [13]. Recently, such a type of molecule 6-(3-methyltriaz-1-en-1-yl)-1*H*-benzo[de]isoquinoline-1,3(2*H*)-dione, EG22 (**8a**, Scheme 2), was designed to behave as an inhibitor of a DNA repair protein termed “poly(adenosine diphosphate ribose) polymerase” (PARP) and a DNA alkylating agent. EG22 (**8a**) was successfully synthesized and shown to possess dual PARP and DNA targeting properties [14]. However, its rate of hydrolysis under physiological conditions was too fast and we believed that this could compromise its activity in vivo. Thus, in order to stabilize the combi-molecule, we investigated means to delay its hydrolysis under physiological conditions. Here we report on the unequivocal characterization of 6-(3-acetyl-3-methyltriaz-1-en-1-yl)-1*H*-benzo[de]isoquinoline-1,3(2*H*)-dione, ZSM02 (**9a**), the first prototype of a masked form of EG22 (**8a**), using ^1^H-, ^15^N- and ^13^C-NMR of its isotopically labelled form. We hypothesized that if ZSM02 (**9a**) is a prodrug of EG22 (**8a**), its growth inhibitory potency should be the same as EG22 (**8a**). Therefore, we compared their activity in human breast, colon and brain cancer cell lines.

## 2. Results and Discussion

### 2.1. Stability of EG22 in DMSO

**Figure 1:**
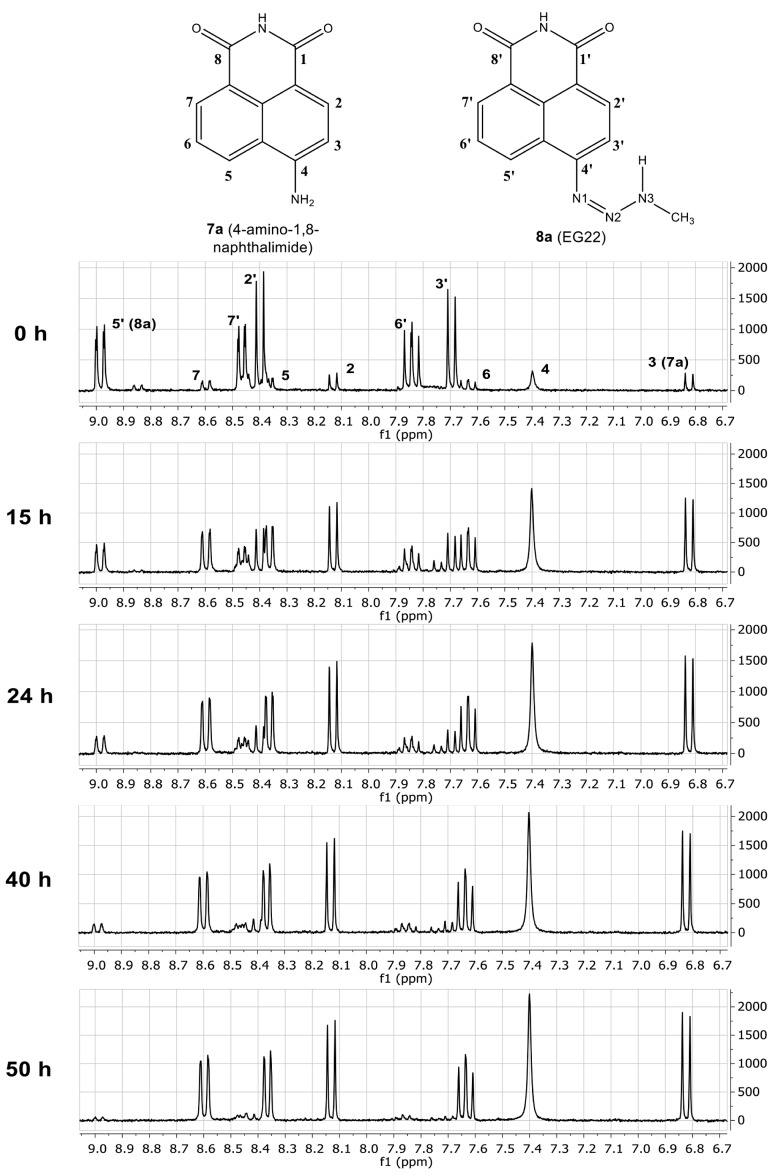
^1^H-NMR spectra of EG22 (**8a**) showing slow conversion to ANI (**7a**) (0–50 h) in non-dried DMSO-*d_6_*.

**Figure 2:**
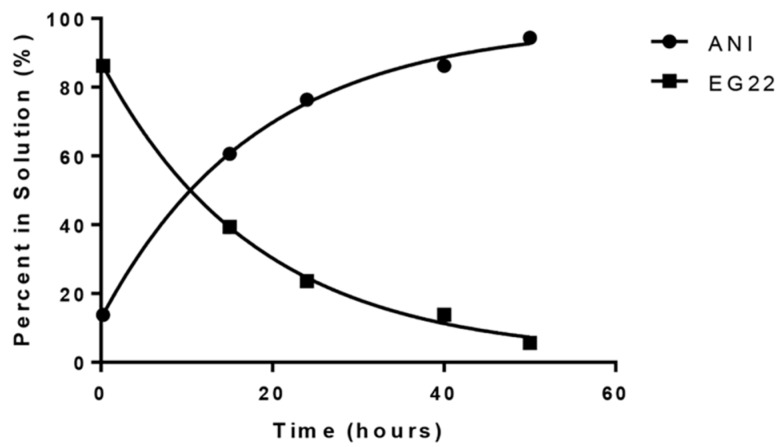
Formation of 4-amino-1,8-naphthalimide, ANI (**7a**) in DMSO over time using ANI/(ANI + EG22) percent peak ratio and decay curve for EG22 (**8a**) in DMSO over time using EG22/(ANI + EG22) percent aromatic peak ratio.

The synthesis of non-isotopically labeled EG22 (**8a**) was reported elsewhere [14]. As shown in Figure 1, the first evidence of its instability was obtained by monitoring the appearance of a peak corresponding to the shielded proton *ortho* to the amino group of 4-amino-1,8-naphthalimide (ANI, **7a**) and the disappearance of a deshielded doublet of the aromatic ring of EG22 (**8a**) in wet DMSO-*d_6_* at room temperature. Indeed, after a two-day period, EG22 (**8a**) was almost fully converted to 4-amino-1,8-naphthalimide (ANI, **7a**) and the decomposition t_1/2_ under these conditions was 12.6 h (Figure 2).

**Scheme 3:**
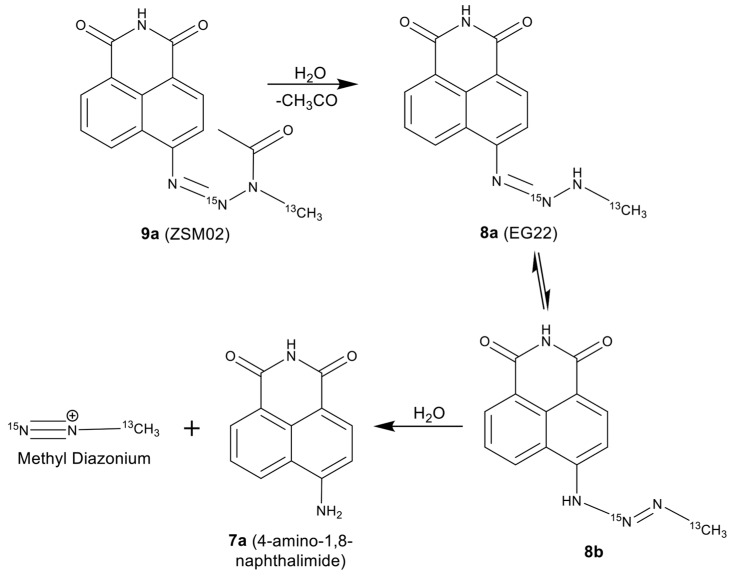
Deacetylation of ZSM02 (**9a**) and protolysis of EG22 (**8a**) to regenerate ANI (**7a**) and create a methyl diazonium species.

The rate determining step of the hydrolysis of monoalkyltriazenes has been shown to be the protolysis of their non-conjugated tautomer [15]. Therefore, we believe that this conversion is catalyzed by trace amount of water in DMSO-*d_6_* and the mechanism of degradation is primarily based on the cleavage of the non-conjugated tautomer **8b** (Scheme 3).

Accordingly, we surmised that the hydrolysis of EG22 (**8a**) could be delayed by acetylating N3, thereby shifting the overall rate determining step to that of the slow cleavage of the N3–CO bond. The fact that EG22 (**8a**) can assume the two tautomeric forms outlined in Scheme 2 and Scheme 3 in organic solutions and since acetylation of each tautomer would lead to different structures of the same mass or resulting from loss of N_2_, a confusing NMR spectrum was expected for the reaction products. Surprisingly, the synthesis led to one major product, the exact structure of which remained elusive. Thus, we undertook an isotopic labeling study involving ^15^N and ^13^C labeling of the 3-methyl-1,2,3-triazene moiety.

### 2.2. Isotopic Labelling and NMR Spectroscopy

As outlined in Scheme 2, the incorporation of the isotopes proceeded by substituting two reactants for their isotopically labelled counterparts in the synthesis of EG22 (**8a**): (a) ^15^N sodium nitrite to be incorporated in the in situ generated diazonium salt and (b) ^13^C methylamine for addition to the latter under basic conditions. Having shown that EG22 (**8a**) can be converted to ANI (**7a**) and considering that it perhaps exists as two tautomers in solution, we expected products resulting from: (i) a direct reaction of the acetyl chloride on the N3 of the triazene moiety, leading to the desired structure **9a**; (ii) an acetylation of the non-conjugated isomer **8b** to produce **9b**; (iii) an acetylation of ANI (**7a**) resulting from the decomposition of EG22 (**8a**) or the loss methyl diazonium from **9b** to give **10b**, or (iv) loss of nitrogen from **9a** and **9b** to give **10a** [16].

**Figure 3:**
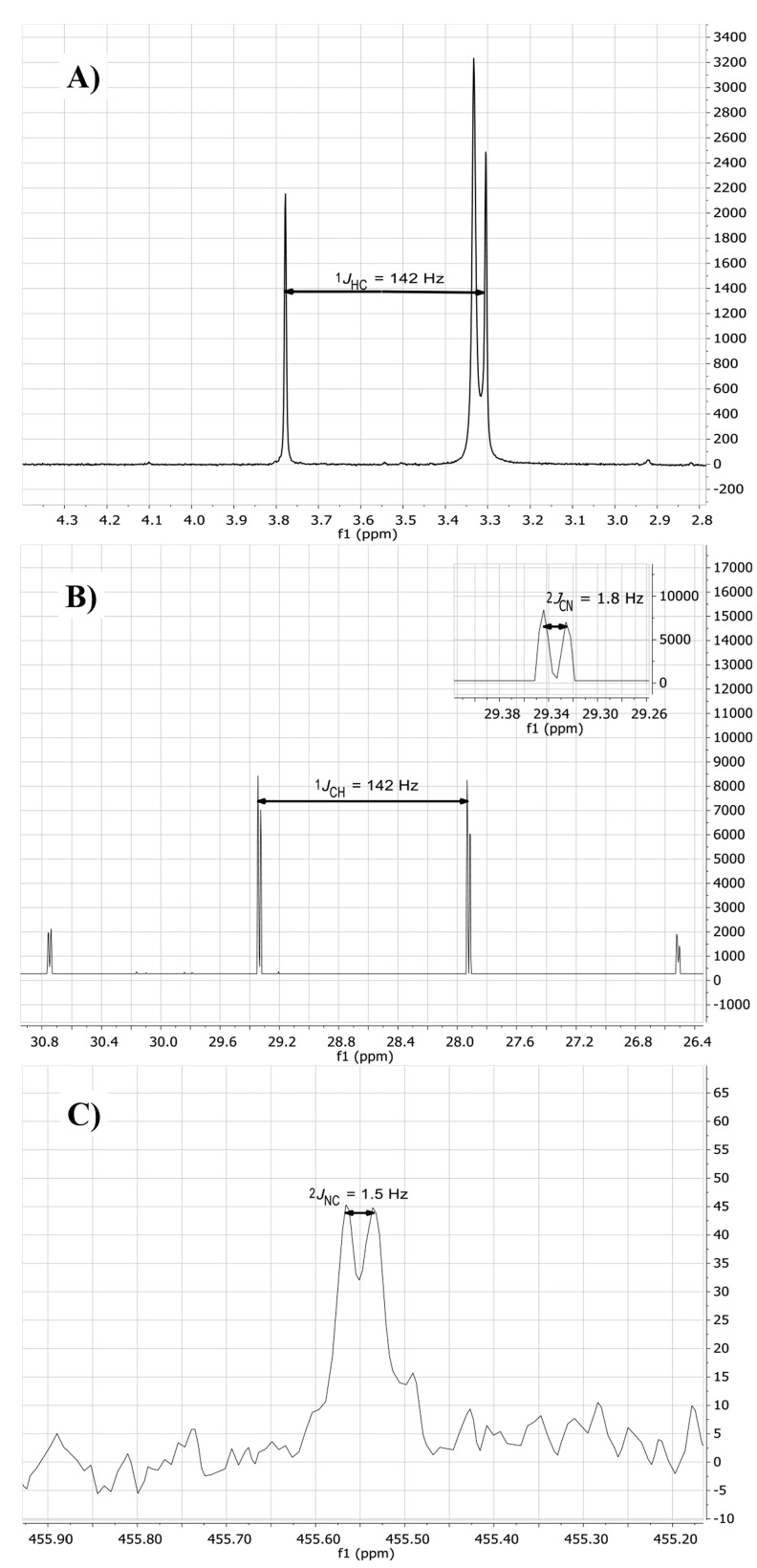
(**A**) ^1^H-NMR spectrum of ^13^C and ^15^N labelled ZSM02 (**9a**) in non-dry DMSO-*d_6_* displaying the ^13^CH_3_ doublet (^1^*J*_CH_ = 142.2 Hz). (**B**) Proton coupled ^13^C-NMR spectrum of ^13^C and ^15^N labelled ZSM02 (**9a**) (^1^*J*_CH_ = 142 Hz and ^2^*J*_CN_ = 1.8 Hz). (**C**) Proton decoupled ^15^N-NMR spectrum of ^13^C and ^15^N labelled ZSM02 (**9a**) (^2^*J*_CN_ = 1.5 Hz).

^1^H-NMR analysis of the product (Figure 3a) showed an interesting coupling pattern for the 3-methyl group, which appeared as a doublet (^1^*J*_HC_ = 142 Hz), indicating that it is coupled with ^13^C [17]. Since we expected this coupling to be consistent with the presence of the ^13^C labeled methyl, we further analyzed the product by ^13^C-NMR.

Interestingly, the results showed that the ^13^C methyl peak appeared at 28.6 ppm, as a quartet of doublets (^1^*J*_CH_ = 142 Hz, ^2^*J*_CN_ = 1.5 Hz) (Figure 3b), indicating that it does not only couple with its directly bound hydrogen but also with the central nitrogen N2 of the triazene chain.

In order to further ascertain the presence of the ^15^N label of the central nitrogen, a full ^15^N-NMR spectrum was acquired in decoupled mode. ^15^N-NMR analysis showed a peak at 455.6 ppm (ammonium scale) or 75 ppm (converted to the nitromethane scale) that showed up as a doublet (^2^*J*_NC_ = 1.8 Hz) as a result of coupling with the isotopically labeled ^13^C (Figure 3c). The observed shift for N2 is in agreement with previous reports by our group showing that the N2 in 1,2,3-triazene containing molecules is in the +70 ppm range [18,19].

**Scheme 4:**
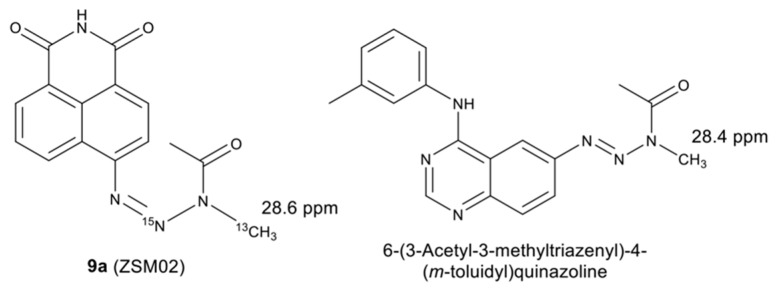
Comparison of ZSM02 (**9a**) to previously synthesized *N*-methylacetyltriazene. The respective N3 methyl shifts are 28.6 ppm and 28.4 ppm*.*

The ^15^N-NMR and its corresponding coupling with the ^13^C-labeled carbon allowed to rule out structures **10a** and **10b** (Scheme 2). Our data is consistent with the presence of an intact 1,2,3-triazene chain in the structure, as in **9a** and **9b**. The ^13^C shift of the ^13^C-labeled methyl group in the non-conjugated tautomer is known to be considerably deshielded (e.g., 54 ppm for 3-methyl-l-*p*-tolyl-triazene) [20]. Our observed shift (28.6 ppm) is significantly more shielded and is consistent with that of a similar *N*-methylacetyltriazene previously synthesized by our group [21]. This allowed to rule out **9b**. As depicted in Scheme 4, in 6-(3-acetyl-3-methyltriazene)-4-(*m*-toluidyl)quinazoline that carries a similar NNN(CH_3_)COCH_3_ moiety, the ^13^C shift of the methyl group (28.4 ppm) is almost identical to that in ZSM02 (**9a**) (28.6 ppm).

### 2.3. HMBC and HSQC Analysis

**Figure 4:**
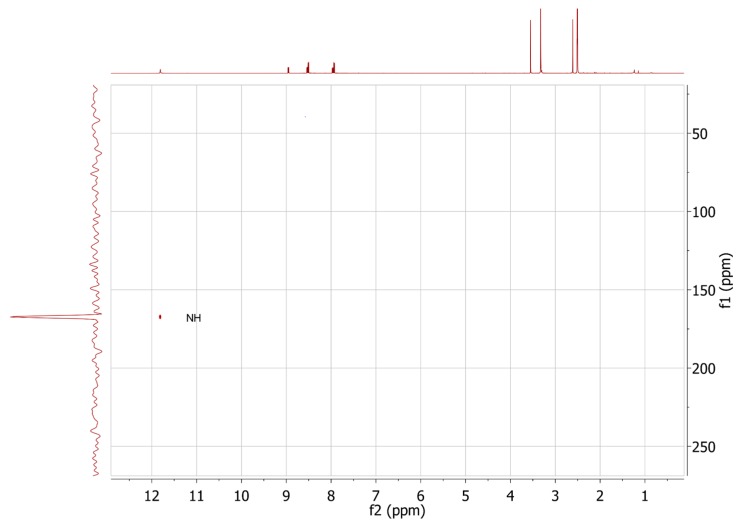
Heteronuclear single quantum coherence (HSQC) experiment showing a sharp peak for the NH of the naphthalimide ring of compound **9a**.

**Figure 5:**
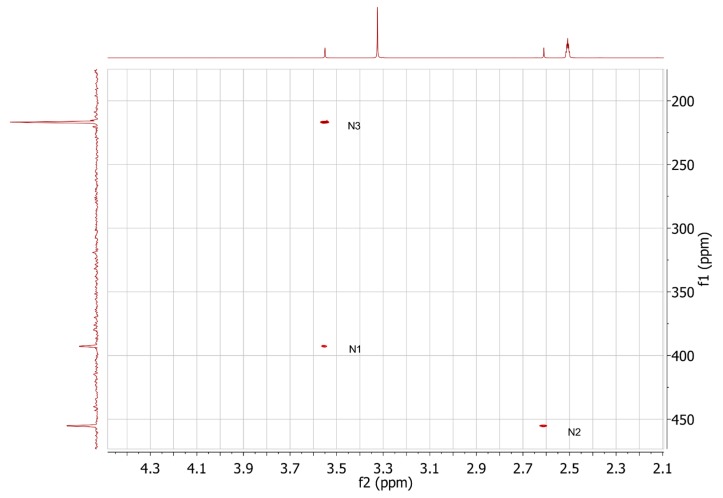
Heteronuclear multiple bond correlation (HMBC) experiment showing peaks corresponding to N1, N2 and N3 of the triazene chain of compound **9a**.

While the ^15^N labeling led to the determination of the presence and chemical shift of the central nitrogen, absence of coupling with the other nitrogens, N1 and N3 in the chain, did not allow us to infer on the presence of the latter two nitrogens. Likewise, the nitrogen of the naphthalimide system could not be detected from the experiment. Therefore, we undertook connectivity studies using heteronuclear multiple bond correlation (HMBC) and heteronuclear single quantum coherence (HSQC) to indirectly detect the latter natural abundance nitrogens. Indeed, HSQC experiment showed a sharp peak for the NH of the naphthalimide ring (Figure 4) at 167 ppm (−213 ppm, nitromethane scale). As depicted in Figure 5, HMBC analysis further confirmed the presence of N1, N2, and N3 at 393.4 (13 ppm), 455.8 (75 ppm), and 217.4 ppm (−163 ppm), respectively. Therefore, these experiments allowed us to confirm the complete nitrogen content of the molecule. Our NMR data in toto confirm that structure **9a** (ZSM02), is the product of the reaction.

### 2.4. Determination of the 3D Structure

**Figure 6:**
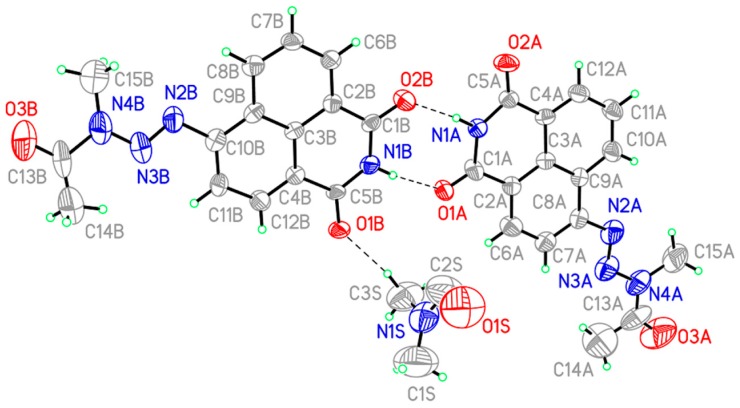
X-ray crystallographic structure of compound **9a**. Non-hydrogen atoms drawn as ellipsoids at the 50% probability level and hydrogen atoms as circles of arbitrary radii.

In order to determine the three-dimensional structure of the molecule, we sought to crystallize f **9a**, but all attempts via conventional methods failed. A dimethylformamide (DMF)/hexane biphasic approach led however to needles that lent themselves to diffraction. The crystal structure was identified as a DMF hemisolvate with two unique molecules of **9a** and one molecule of DMF in the asymmetric unit (Figure 6). As depicted in Figure 4, the results showed that the complete *N*-acetyl triazene moiety was almost completely coplanar with the naphthalimide rings. The least square planes of the acetyl and napthalimide groups were 5.8° and 9.8° for molecules A and B, respectively (as labeled in Figure 4). Acetylation of the nitrogen did not lead to a significant elongation of N2–N3 bond (N4A–N3A: 1.36(2) Å; N4B–N3B: 1.34(2) Å), which is consistent with the observed coplanarity and perhaps the electron donating character of the *N*-acetyltriazene moiety. Indeed, NMR analysis showed that the proton attached to C11B (Figure 4) is more shielded than the one at C8B in naphthalimide ring. Reported lengths for non-acetylated open-chain triazenes are in the range of 1.30 Å [22].

### 2.5. Growth Inhibitory Potency

**Figure 7:**
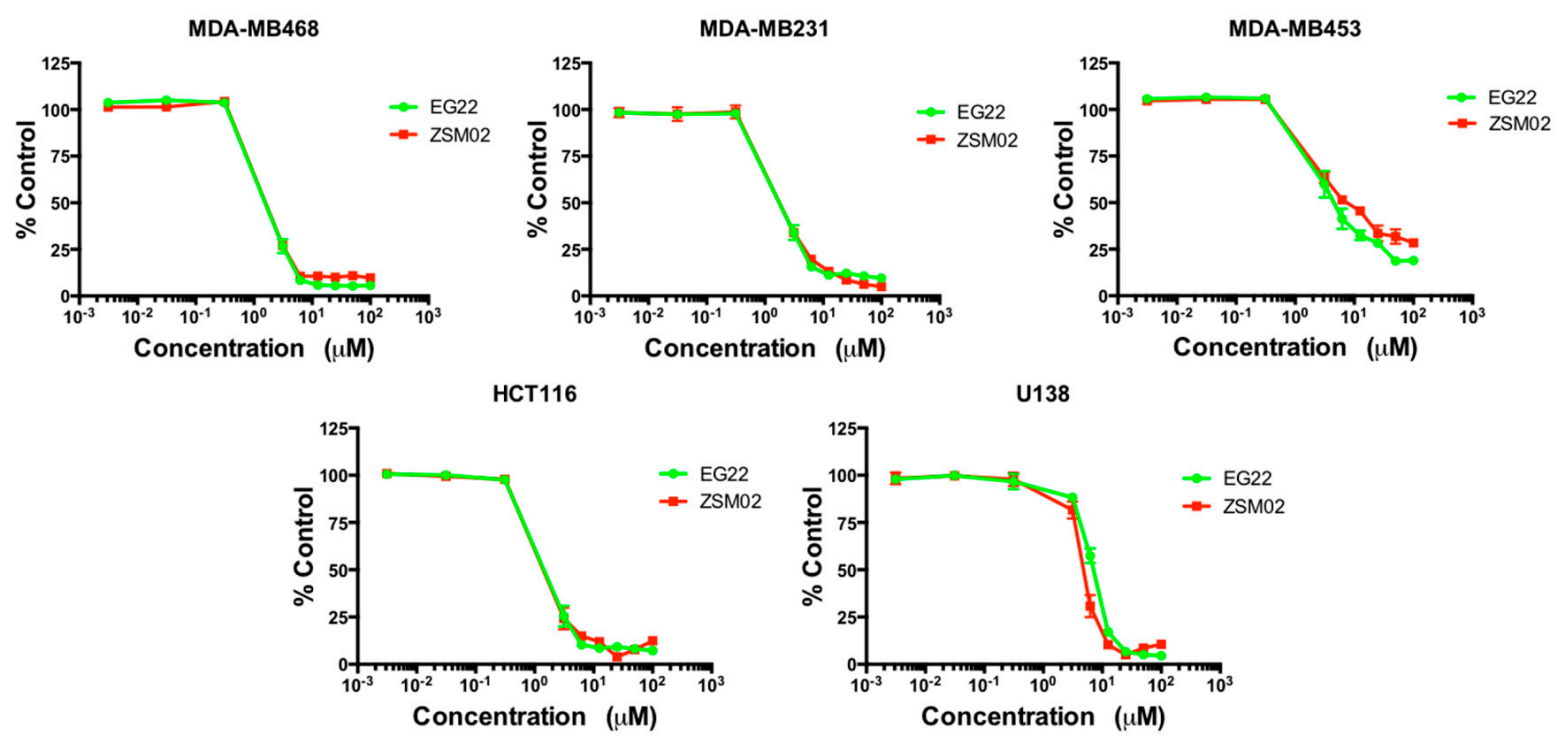
Growth inhibition activity of compounds **8a** (EG22) and **9a** (ZSM02).

Having ascertained the structure of ZSM02 (**9a**), we further analyzed its effect on cell growth in comparison with its precursor, EG22 (**8a**). We surmised that if ZSM02 (**9a**) is converted into EG22 (**8a**) intracellularly, their growth inhibitory profile should be similar. Indeed, as shown in Figure 7, using the Sulforhodamine B (SRB) growth inhibitory assay [23], we confirm that ZSM02 (**9a**) and EG22 (**8a**) exhibit similar growth inhibitory profiles. Further work on the mechanism of action of both molecules is reported elsewhere [14].

## 3. Material and Methods

### 3.1. General Information

4-Amino-1,8-naphthalimide (ANI) was purchased from Ark Pharm (Arlington, Heights, IL, USA). All other chemicals were purchased from Sigma-Aldrich (Oakville, CA, USA).

### 3.2. Chemical Synthesis

*6-(3-Methyltriaz-1-en-1-yl)-1H-benzo[de]isoquinoline-1,3(2H)-dione* (**8a**): The methyltriazene compound **8a** was synthesized as described in Scheme 2. Briefly, 4-amino-1,8-naphthalimide (ANI, **7a**) (1 eq., 0.236 mmol) was dissolved in concentrated trifluoroacetic acid and was cooled to −5 °C for 15 min. The ^15^N labeled sodium nitrite (2 eq., 0.472 mmol) in a clear solution was then added dropwise. Once diazotized, ^13^C labelled methylamine hydrochloride (3 eq., 0.708 mmol) was dissolved in water and added slowly dropwise thereafter. Upon reaction completion, the solution was neutralized with a saturated solution of sodium bicarbonate and left to precipitate for an hour. The mixture was then filtered and the precipitate collected and dried. ^1^H-NMR (300 MHz, DMSO-*d_6_*) δ ppm 11.61 (s, 1H, NH), 11.44 (q, 1H, *J* = 3.6 Hz, NHCH_3_), 8.97 (dd, 1H, *J* = 8.4 Hz, 0.9Hz, ArH), 8.46 (dd, 1H, *J* = 7.2 Hz, 1.2 Hz, ArH), 8.39 (d, 1H, *J* = 8.1 Hz, ArH), 7.83 (t, 1H, *J* = 8.0 Hz, ArH), 7.69 (d, 1H, *J* = 8 Hz, ArH), 3.26 (dd, 3H, *J* =139.3 Hz, 4.2 Hz, NH^13^CH_3_).

*6-(3-Acetyl-3-methyltriaz-1-en-1-yl)-1H-benzo[de]isoquinoline-1,3(2H)-dione* (**9a**): The acetylated compound **9a** was synthesized as described in Scheme 2. Briefly, 3 mL of anhydrous pyridine was flash frozen using liquid nitrogen. Once completely frozen, acetic anhydride (10 eq., 1.97 mmol) was introduced and flash frozen using liquid nitrogen. A total of 50 mg of **8a** (EG22) in Scheme 2 (1 eq, 0.197 mmol) was added as a powder. The reaction was allowed to reach a temperature of −5 °C for 30 min and then reach room temperature slowly for 2 h. Once the reaction was complete, the pyridine was azeotroped with toluene. The resulting solid was collected and dried. ^1^H-NMR (300 MHz, DMSO-*d_6_*) δ ppm 11.83 (s, 1H, NH), 8.96 (dd, 1H, *J* = 8.4 Hz, 0.8 Hz, ArH), 8.54 (dd, 1H, *J* = 7.2 Hz, 1.2 Hz, ArH), 8.51 (d, 1H, *J* = 8.0 Hz, ArH), 7.96 (t, 1H, *J* = 8.0 Hz, ArH), 7.94 (d, 1H, *J* = 8 Hz, ArH), 3.54 (d, 3H, *J* = 142.2 Hz, N^13^CH_3_), 2.60 (s, 3H, COCH_3_). ^13^C-NMR (75.4 MHz, DMSO-*d_6_*) δ ppm 172.96, 163.71, 148.35, (2C) 130.56, 130.53, 129.78, 129.69, 127.72, 127.57, 122.74, 122.18, 114.33, 28.63 (qd, *J* = 142 Hz, 1.8 Hz, ^15^NN^13^CH_3_) and 22.03. ^15^N-NMR (50.7 MHz, DMSO-*d_6_*) δ ppm 455.55 (d, *J* = 1.5 Hz, ^15^NN^13^C). ESI *m/z* 297 (MH^−^).

### 3.3. NMR Acquisition

The ^1^H- and ^13^C-NMR spectra were acquired at ambient temperature on a Mercury 300 spectrometer (Varian/Agilent, Palo Alto, CA, USA) equipped with an automated triple broadband (ATB) probe. Concentration of samples were 1 mg/mL in DMSO-*d_6_*. The relaxation delay was 1 s after a 45 degrees pulse and acquisition time of 1042 s. For the ^1^H-NMR spectrum, 256 scans were collected and for the ^13^C coupled spectrum 6800 scans were collected. Both ^13^C spectra were acquired with nuclear overhauser effect (NOE). The spectral width was 18,832 Hz, 39,248 points were collected and zero filled to 256K points before Fourier transformation for a digital resolution of 0.14 Hz. No apodization was used.

The ^15^N-NMR spectrum was obtained at ambient temperature on a Varian/Agilent VNMRS 500 spectrometer (Palo Alto, CA, USA) equipped with a dual broadband probe. Concentration of the sample was 5 mg/mL in DMSO-*d_6_* and was referenced using ^15^N ammonia as an external standard and for conversion to the nitromethane scale the following equation was used: δ(nitromethane) = δ (ammonia) – 380.3. The relaxation delay was 3 s after a 20-degree pulse and acquisition time of 1.6 s. A total of 1064 scans were collected. The spectrum was acquired with NOE. The spectral width was 25,000 Hz, 80,004 points were collected and zero-filled to 512K points before Fourier transformation for a digital resolution of 0.10 Hz.

All spectra of the non-isotopically labeled compounds were acquired on an AVIIIHD spectrometer (Bruker, Faellanden, Switzerland) operating at a ^1^H frequency of 500.3 MHz using a BBFO + SmartProbe (Bruker, Faellanden, Switzerland). Around 2 mg of ZSM02 (**9a**) were dissolved in 1 g DMSO-*d*_6_. The ^1^H spectrum was acquired in 72 scans using an acquisition time of 3.7 s and a recycle delay of 1 s. The ^13^C spectrum was acquired using power gated WALTZ decoupling in 6144 scans with a recycle delay of 2 s (total experimental time 5.5 h). The homonuclear correlation spectroscopy (COSY) spectrum was acquired in 20 min using a spectral width of 10 ppm in each dimension, 2048 points in the direct dimension, and 256 points in the indirect dimension. Two ^13^C-HSQC spectra were acquired, one with a spectral width in the indirect dimension of 165 ppm and 64 points (4 min) and the other centered on 129.5 ppm with a spectral width of 8 ppm and 512 points (30 min). The ^13^C HMBC spectrum was acquired using a spectral width in the indirect dimension of 65 ppm centered at 143 ppm using 384 points (one hour). The ^15^N-HSQC spectrum was acquired in 1.25 h using optimization for a 90 Hz *J* coupling. The ^15^N-HMBC spectrum was acquired in 8.5 h using 16 scans per increment and 512 increments, in 8.5 h, with delay times optimized for a 1.6 Hz *J* coupling.

### 3.4. X-ray Crystallography

Data collection was performed on a Bruker D8 Venture equipped with a Photon 100 area detector (Bruker-AXS, Madison, WI, USA). Yellow, crystalline needles were isolated by inspection under microscope. Although the crystals were well-formed and transparent, they showed signs of polycrystallinity including uneven extinction under polarized light and the ability to bend. Diffraction was unexpectedly weak for crystals of their size, and cooling the crystals to low temperature appeared to result in even weaker diffraction. This behavior suggested the crystals may have been aggregates of much smaller crystallites which remained fairly well aligned at room temperature but lost their alignment under the strain of thermal contraction on cooling. Ultimately, the best data was collected using graphite monochromated Mo-Kα radiation from a microfocus source on a crystal measuring 0.22 × 0.08 × 0.01 mm using shutterless scans at a rate of 120 s/degree. A full hemisphere of unique data was collected out to 0.80 Å resolution using scans about the omega and phi axes. Unit cell determination, data collection, data reduction, and absorption correction were performed using the Bruker Apex3 software suite (version 2015-R7; Bruker-AXS, Madison, WI, USA).

The structure of **9a** was solved and its space group determined by the iterative dual space approach implemented in the program SHELXT [24]. The absolute configuration of the crystal structure could not be determined from Mo Kα data. Non-hydrogen atoms were refined anisotropically by full-matrix least squares refinement against F^2^. Hydrogen atoms were placed in calculated positions, and their coordinates and thermal parameters were constrained to ride on the carrier atom. Least squares refinement was done using SHELX v.2014 [25] implemented in the Bruker SHELXTL software suite [26].

Diffraction from this crystal becomes essentially indistinguishable from noise at resolutions beyond 1 Å, so the precision on bond distances is low (approximately 0.02 Å). The structural features of interest—the 3-D configuration of the molecule and the distinction between single, double, and aromatic bonds by length—can all still be unambiguously detected at this resolution. Additionally, most of the reflections lie in the 1.0–0.8 Å resolution range, so the global values for R_int_, wR_2_ are high while the ratio of observed to unique reflections is low. Values that are constrained to resolutions containing observed reflections, such as R_1_, are in acceptable ranges. While these deficiencies could be solved by using a larger crystal or a more powerful X-ray source, the refinement presented here represents the limit of what could be achieved with available facilities and is chemically reasonable and fully consistent with other experimental characterization.

Crystal data for **9a**·0.5DMF (ZSM02, **9a**): C_16.5_H_15_N_4.5_O_3.5_ (Mw = 332.33 g/mol), monoclinic, space group *Pc*, *a* = 13.856(3) Å, *b* = 4.9842(9) Å, *c* = 23.836(5) Å, β = 100.651(6)°, α = γ = 90°, V = 1617.8(5) Å^3^, Z = 4, T = 298(2) K, λ(Mo Kα) = 0.71073 Å, Dcalc = 1.364 mg/m^3^, F(000) = 694, independent reflections 24669/6617 (R_int_ = 0.2463), 2.494° < 2*θ* < 26.435°, the final R_1_ was 0.1434 (I > 2σ(I)) and wR_2_ was 0.3900 (all data).

The supplementary crystallographic data for compound **9a** can be obtained in Supplementary Materials. The Crystallography Open Database contains the supplementary crystallographic data for this paper. These data can be obtained free of charge at http://www.crystallography.net/cod/search.html (COD no. 3000124).

### 3.5. Cell Culture

HCT116 colon cancer cell line was kindly provided by Moulay Alaoui-Jamali (Segal Cancer Centre and Lady Davis Institute for Medical Research, Jewish General Hospital, Montreal, QC, Canada). The MDA-MD468 breast cancer cell line was bought from the ATCC (Manassas, VA, USA). MDA-MB453 and MDA-MB231 were a generous gifts of Suhad Ali (the Research Institute of the McGill University Health Centre, Montreal, QC, Canada). U138 glioblastoma cell lines were kindly given by Siham Sabri (the Research Institute of the McGill University Health Centre, Montreal, QC, Canada). Media preparation was supplemented with 10% fetal bovine serum (FBS), 10 mM HEPES, gentamicin sulfate, amphotericin B and ciprofloxacin. All the reagents used in the preparation of the media were purchased from Wisent Inc. (St-Bruno, QC, Canada). The cells were grown in a humidified incubator with a stable temperature of 37 °C and CO2 level of 5%.

### 3.6. Growth Inhibition Assay

Cells were plated in 96-well plates (Corning Inc., Corning, NY, USA) at 3000–10,000 cells/well in 100 μL medium/well. They were then treated, 24 h later, with a wide range of drug concentrations (0.0031 μM to 800 μM). The treatment was done in triplicate for 5 days in the incubator. Following the drug treatment, the cells were fixed with 50 μL per well of cold TCA (50%) for 2 h at 4 °C, rinsed, dried well and stained with 50 μL sulforhodamine B (SRB) (0.4 g/100 mL). Subsequently, the SRB was rinsed with 1% acetic acid, and allowed to air-dry overnight. Finally, the dye was solubilized with Tris base (10 mM, pH 10–10.5). Absorbance readings of the solubilized dye were recorded on a ELx808 microplate reader, BioTek (Winooski, VT, USA) at an optical density of 492 nm. The results were analyzed using GraphPad Prism software (GraphPad Software, La Jolla, CA, USA) to derive a dose-response curves and the IC50. Each experiment was carried out four times, in triplicate.

## 4. Conclusions

We successfully acetylated the 1,2,3-methyl triazene **8a**. Subsequently, we unequivocally assigned the structure of the resulting compound **9a** (ZSM02), using ^15^N isotopic labeling, HMBC, HSQC and X-ray crystallography. Finally, a biological assay showed similar growth inhibitory potency for both compounds **8a** (EG22) and its acetylated form, **9a**. These results in toto suggest that **9a** could be a prodrug of **8a.** A detailed study on the hydrolysis of **8a** and **9a** under physiological conditions and on their biological effects as combi-molecules, is reported elsewhere [14].

## Supplementary Materials

The following are available online: Crystal structure in CIF format of compound **9a**.
